# A Case of Superior Mesenteric Artery Syndrome in a Patient With Acute-Onset Symptoms Managed Conservatively

**DOI:** 10.7759/cureus.98328

**Published:** 2025-12-02

**Authors:** Abhishek Shrestha, Kehinde Adekeye, Rohith Keezhath

**Affiliations:** 1 Internal Medicine, Medway NHS Foundation Trust, Gillingham, GBR; 2 Acute Medicine, Medway NHS Foundation Trust, Gillingham, GBR

**Keywords:** conservative vs. surgical management, persistent vomiting, small-bowel obstruction, superior mesenteric artery (sma), superior mesenteric artery syndrome, wilkie’s syndrome

## Abstract

Superior mesenteric artery (SMA) syndrome is a rare aetiology of duodenal obstruction, typically presenting with chronic abdominal pain, nausea, vomiting, and weight loss. The syndrome results from a decreased angle between the SMA and the abdominal aorta, which compresses the third portion of the duodenum and obstructs the duodenal lumen. This report describes a 31-year-old male who presented with acute-onset vomiting and epigastric pain and was diagnosed with SMA syndrome. Although SMA syndrome classically presents with chronic symptoms, this case demonstrates an acute presentation in an otherwise healthy young adult. This case highlights the necessity of including SMA syndrome in the differential diagnosis of acute abdominal symptoms, even in patients without a chronic history, as early recognition and conservative treatment may prevent surgical procedures.

## Introduction

Superior mesenteric artery (SMA) syndrome, also known as Wilkie’s syndrome, is a rare gastrointestinal disorder resulting from a reduced angle between the SMA and the abdominal aorta. This anatomical change compresses the third portion of the duodenum, resulting in mechanical obstruction of the duodenal lumen [1]. SMA syndrome is quite rare, with an incidence of 0.1-0.3% [2]. Patients typically present with chronic symptoms such as postprandial epigastric pain, nausea, vomiting, and weight loss [3-6]. These symptoms can have intermittent exacerbations depending on the cause and grade of duodenal compression. In rare instances, symptoms may present acutely and progress to upper abdominal ileus [4]. The prevalence of SMA syndrome varies by symptom onset: acute cases occur at a rate of 0.0108-0.0520 per 1,000 admissions, and chronic cases at a rate of 0.965 per 1,000 admissions [4]. Because symptoms are often vague and non-specific, misdiagnosis is common, and early diagnosis remains difficult [1]. Limited clinician awareness frequently leads to delayed diagnosis, prolonged disease course, and delayed imaging [4].

## Case presentation

The patient was a 31-year-old, thin-built male with a history of attention-deficit/hyperactivity disorder, daily vaping, and previous alcohol use who presented to same-day emergency care with a four-day history of acute-onset gastrointestinal symptoms. His illness began with the sudden onset of epigastric pain and several episodes of loose stools on day one. The loose stools persisted only for that initial day, after which he reported no further bowel movements. Within several hours of the onset of abdominal pain and diarrhoea, he developed nausea and vomiting, which progressively worsened over the next three days. Initially occurring a few times per day, the vomiting increased to five to ten bilious, non-bloody episodes daily by the time of presentation. His epigastric pain persisted throughout the four days, described as a constant dull ache without radiation. He denied any fever, chills, respiratory symptoms, headache, loss of consciousness, gastrointestinal bleeding, or abdominal distension. He reported no similar prior episodes. The patient did not experience unintentional weight loss, appetite reduction, or alterations in eating patterns. His weight had remained stable over the past 6-12 months, and he reported no recent illness or lifestyle modifications that could account for catabolic weight loss.

On examination, the patient was alert and oriented, haemodynamically stable, with tenderness localised to the epigastric and right hypochondriac regions. Laboratory investigations demonstrated normal white blood cell count, haemoglobin, urea and electrolytes, liver enzymes, C-reactive protein (4.9 mg/L), troponin, and venous blood gas lactate (1.1 mmol/L). The electrocardiogram revealed a normal sinus rhythm.

**Figure 1:**
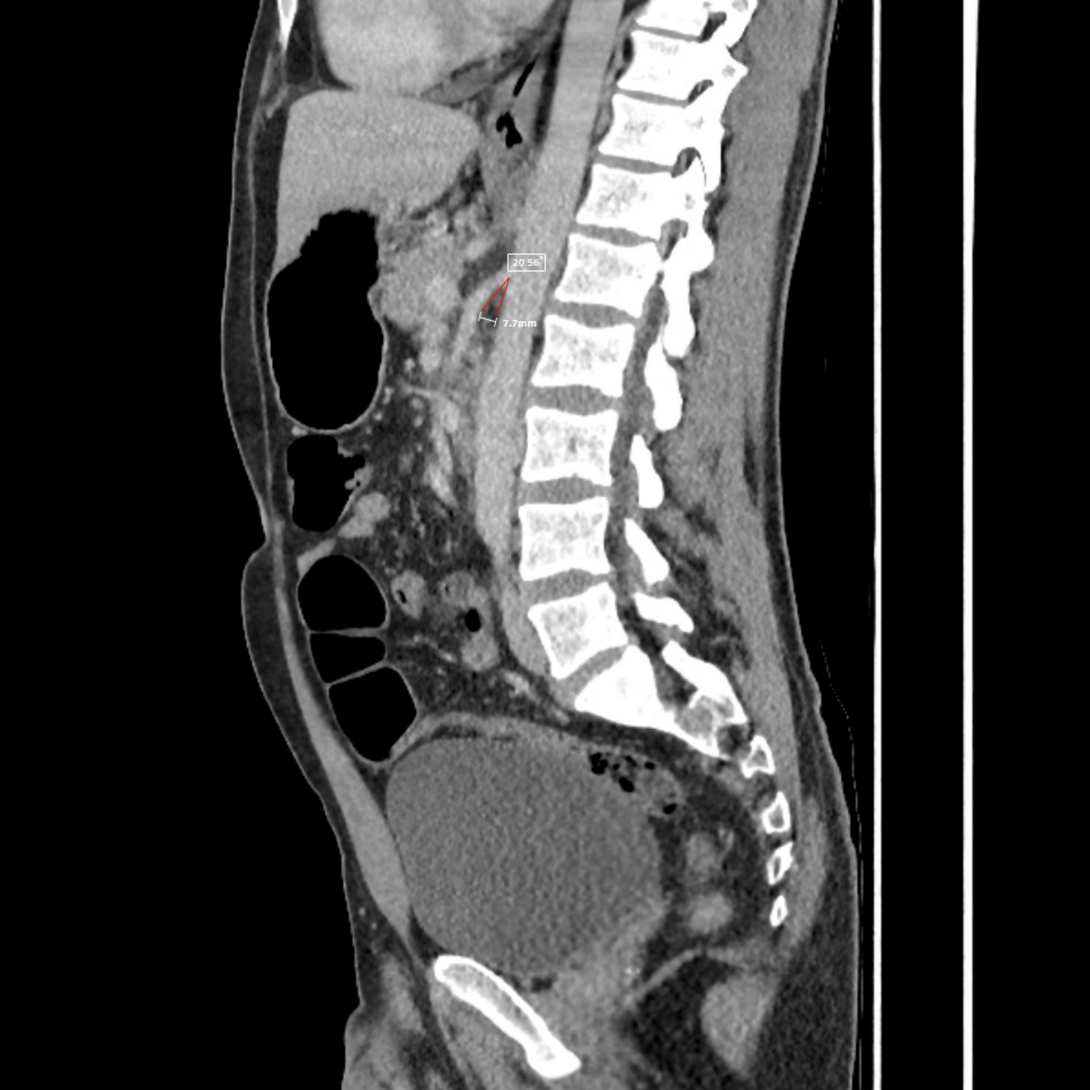
CT of the abdomen and pelvis with contrast, sagittal slice, showing a decreased aorto-mesenteric angle of 20 degrees (measured) and decreased aorto-mesenteric distance of 7.7 mm, suggestive of superior mesenteric artery syndrome.

A contrast-enhanced CT scan of the abdomen and pelvis was performed to evaluate for acute abdominal pathology. Imaging demonstrated dilation of the stomach and proximal duodenum with fluid accumulation up to the third part and compression of the fourth part between the SMA and the aorta with a reduced aorto-mesenteric angle (20 degrees) and aorto-mesenteric distance (7.7 mm) (Figure 1). These findings were consistent with SMA syndrome.

Other mechanical and inflammatory causes were systematically excluded. Normal inflammatory markers and white blood cell count did not support viral or infectious gastroenteritis. The absence of fever, resolution of loose stools before presentation, and no evidence of small-bowel dilation or bowel wall oedema on imaging made transient ileus unlikely. CT imaging revealed no mesenteric inflammation, pancreatitis, gallbladder pathology, or obstructive lesions.

On admission, he was haemodynamically stable. After surgical consultation, conservative management was advised, with instructions for nasogastric decompression if symptoms recurred. He received intravenous fluids and antiemetics, with marked improvement in symptoms within 12-18 hours. His vomiting resolved completely, abdominal pain subsided, and bowel activity gradually returned. His diet was advanced from clear liquids to soft consistency over the next 24 hours without recurrence of symptoms. The patient remained clinically well and was discharged after a total hospital stay of approximately 24-36 hours.

At discharge, the patient was advised to consume small, frequent meals and to engage in posture therapy, including lying in the left lateral decubitus position after eating to facilitate duodenal passage and reduce recurrence of symptoms. He was also instructed to return if vomiting or abdominal pain recurred. The patient was further advised to increase daily caloric intake as part of nutritional support to promote weight gain.

## Discussion

The normal angle between the aorta and the SMA ranges from 25 to 60 degrees, with an aortomesenteric distance of approximately 10 to 28 mm. When this angle decreases to less than 25 degrees, and the distance narrows to 2-8 mm, characteristic signs and symptoms of SMA syndrome develop [2].

SMA syndrome is broadly classified as either congenital or acquired [7]. Congenital causes include an abnormally high insertion or a shortened ligament of Treitz, as well as a low origin of the SMA [2,6]. Acquired forms are primarily associated with loss of retroperitoneal and mesenteric fat due to weight loss, which shortens the distance between the aorta and the duodenum, resulting in duodenal obstruction [6]. Acquired aetiologies are linked to medical conditions that induce a catabolic state or rapid weight loss, such as malignancy, malabsorption syndromes, acquired immunodeficiency syndrome, trauma, and burns [2,4]. Additionally, patients undergoing scoliosis surgery, bariatric surgery, ileoanal pouch anastomosis, or oesophagectomy are at increased risk of developing SMA syndrome due to rapid postoperative weight loss [3,4].

Most patients present with chronic abdominal symptoms that intermittently worsen, depending on the underlying cause and severity of duodenal compression [4]. The most common clinical finding is intermittent or postprandial abdominal pain, occurring in 59% to 81% of cases. This is typically followed by vomiting, nausea, and anorexia, which may result in weight loss [4]. Less common symptoms include oesophageal reflux, early satiety accompanied by a sensation of fullness due to increased gastroduodenal transit time, and gastric distension [2,3,4,7].

Several imaging modalities are available, including plain abdominal radiographs that demonstrate gastroduodenal distention, upper gastrointestinal barium studies that reveal anti-peristaltic flow, and CT scans that show a decreased angle and distance between the aorta and the SMA [8]. Previously, ultrasonography of the abdomen with Doppler was used to measure the aortomesenteric angle; however, contrast-enhanced CT of the abdomen is now considered the diagnostic standard [9]. Additional studies, such as MRI and ultrasound, may provide supplementary information and help confirm the diagnosis [8].

A detailed patient history and relevant imaging findings should strongly increase the clinical suspicion of SMA syndrome. Delayed diagnosis may result in serious complications, including electrolyte imbalance, catabolic wasting, peritonitis, and gastric perforation [7].

Initial management is conservative and includes fluid resuscitation, electrolyte repletion, nasogastric or jejunal tube decompression, followed by gradual dietary advancement with nutritional support as needed. It has a success rate of approximately 70-80% in uncomplicated cases. Patients with cachexia may require jejunal or parenteral nutrition to promote mesenteric fat pad accumulation, thereby increasing the aortomesenteric distance and angle [4,5,8]. If conservative management fails, surgical interventions such as gastrojejunostomy, duodenojejunostomy, and Strong’s operation have been utilised to resolve or bypass duodenal compression [4]. Duodenojejunostomy, in particular, demonstrates a 90% success rate and is considered the preferred procedure as it provides immediate relief and is associated with minimal postoperative complications [5,7].

This case is atypical because the patient presented with acute symptoms in the absence of significant weight loss or chronic illness, highlighting the importance of considering SMA syndrome even in patients without classic risk factors. Persistent bilious vomiting and localised epigastric pain prompted expedited imaging, which confirmed SMA syndrome. Early diagnosis allowed conservative management with intravenous hydration and antiemetic therapy, leading to complete symptom resolution. On discharge, the patient was advised to progressively increase daily caloric intake to promote weight gain and restore the mesenteric fat pad. These findings suggest that acute presentations, when promptly identified, may respond well to conservative treatment and may not require surgery.

## Conclusions

SMA syndrome is a rare clinical condition characterised by atypical symptoms that are frequently overlooked. Delayed recognition can result in postponed management and increased morbidity due to small bowel obstruction. Clinicians should improve their understanding of SMA syndrome and include it in the differential diagnosis for patients with postprandial abdominal pain, anorexia, vomiting, or weight loss. This case demonstrates that patients can present with acute-onset symptoms in the absence of a chronic history or significant weight loss. Detailed history-taking and imaging, such as abdominal CT, enabled prompt diagnosis and effective conservative management. These findings underscore the importance of thorough clinical evaluation and early diagnostic investigations to improve patient outcomes and reduce the need for surgical intervention.
